# Publisher Correction: A multi-ethnic epigenome-wide association study of leukocyte DNA methylation and blood lipids

**DOI:** 10.1038/s41467-021-24600-z

**Published:** 2021-07-06

**Authors:** Min-A Jhun, Michael Mendelson, Rory Wilson, Rahul Gondalia, Roby Joehanes, Elias Salfati, Xiaoping Zhao, Kim Valeska Emilie Braun, Anh Nguyet Do, Åsa K. Hedman, Tao Zhang, Elena Carnero-Montoro, Jincheng Shen, Traci M. Bartz, Jennifer A. Brody, May E. Montasser, Jeff R. O’Connell, Chen Yao, Rui Xia, Eric Boerwinkle, Megan Grove, Weihua Guan, Pfeiffer Liliane, Paula Singmann, Martina Müller-Nurasyid, Thomas Meitinger, Christian Gieger, Annette Peters, Wei Zhao, Erin B. Ware, Jennifer A. Smith, Klodian Dhana, Joyce van Meurs, Andre Uitterlinden, Mohammad Arfan Ikram, Mohsen Ghanbari, Deugi Zhi, Stefan Gustafsson, Lars Lind, Shengxu Li, Dianjianyi Sun, Tim D. Spector, Yii-der Ida Chen, Coleen Damcott, Alan R. Shuldiner, Devin M. Absher, Steve Horvath, Philip S. Tsao, Sharon Kardia, Bruce M. Psaty, Nona Sotoodehnia, Jordana T. Bell, Erik Ingelsson, Wei Chen, Abbas Dehghan, Donna K. Arnett, Melanie Waldenberger, Lifang Hou, Eric A. Whitsel, Andrea Baccarelli, Daniel Levy, Myriam Fornage, Marguerite R. Irvin, Themistocles L. Assimes

**Affiliations:** 1 Department of Epidemiology, University of Michigan School of Public Health, Ann Arbor, MI USA; 2 Department of Medicine, Stanford University School of Medicine, Stanford, CA USA; 3 Population Sciences Branch, National Heart, Lung, and Blood Institute, National Institutes of Health, Bethesda, MD USA; 4 Department of Cardiology, Boston Children’s Hospital, Boston, MA USA; 5 Research Unit Molecular Epidemiology, Institute of Epidemiology, Helmholtz Zentrum München, German Research Center for Environmental Health, Munich, Germany; 6 Institute of Epidemiology, Helmholtz Zentrum München, German Research Center for Environmental Health, Munich, Germany; 7 Department of Epidemiology, University of North Carolina, Chapel Hill, NC USA; 8 Hebrew SeniorLife, Beth Israel Deaconess Medical Center, Harvard Medical School, Boston, MA USA; 9 The Brown Foundation Institute of Molecular Medicine, McGovern Medical School, The University of Texas Health Science Center at Houston, Houston, TX USA; 10 Department of Epidemiology, Erasmus MC University Medical Center, Rotterdam, The Netherlands; 11 Department of Epidemiology, School of Public Health, University of Alabama at Birmingham, Birmingham, AL USA; 12 Department of Medical Sciences, Molecular Epidemiology and Science for Life Laboratory, Uppsala University, Uppsala, Sweden; 13 Department of Epidemiology, Tulane University School of Public Health and Tropical Medicine, New Orleans, LA USA; 14 Department of Twin Research and Genetic Epidemiology, School of Life Course Sciences, King’s College London, London, UK; 15 GENYO, Center for Genomics and Oncological Research Pfizer/University of Granada/Andalusian Regional Government, Granada, Spain; 16 Department of Population Health Sciences, University of Utah, Salt Lake City, UT USA; 17 Cardiovascular Health Research Unit, Departments of Medicine and Biostatistics, University of Washington, Seattle, WA USA; 18 Cardiovascular Health Research Unit, Department of Medicine, University of Washington, Seattle, WA USA; 19 Division of Endocrinology, Diabetes, and Nutrition, University of Maryland School of Medicine, Baltimore, MD USA; 20 Program for Personalized and Genomic Medicine, University of Maryland School of Medicine, Baltimore, MD USA; 21 School of Public Health, University of Texas Health Science Center at Houston, Huston, TX USA; 22 Division of Biostatistics, School of Public Health, University of Minnesota, Minneapolis, MN USA; 23 Institute of Genetic Epidemiology, Helmholtz Zentrum München, German Research Center for Environmental Health, Neuherberg, Germany; 24 IBE, Faculty of Medicine, LMU Munich, Munich, Germany; 25 Institute of Medical Biostatistics, Epidemiology and Informatics (IMBEI), University Medical Center, Johannes Gutenberg University, Mainz, Germany; 26 Institute of Human Genetics, Helmholtz Zentrum München, German Research Center for Environmental Health, Munich, Germany; 27 Institute of Human Genetics, Technical University Munich, Munich, Germany; 28 Survey Research Center, Institute for Social Research, Ann Arbor, MI USA; 29 Department of Internal Medicine, Rush University Medical Center, Chicago, IL USA; 30 Department of Internal Medicine, Erasmus MC University Medical Center, Rotterdam, The Netherlands; 31 School of Biomedical Informatics, University of Texas Health Science Center at Houston, Houston, TX USA; 32 Department of Epidemiology and Biostatistics, School of Public Health, Peking University Health Science Center, Beijing, China; 33 Institute for Translational Genomics and Population Sciences, Los Angeles Biomedical Research Institute, and Department of Pediatrics, Harbor-UCLA Medical Center, Torrance, CA USA; 34 HudsonAlpha Institute for Biotechnology, Huntsville, AL USA; 35 Department of Human Genetics, David Geffen School of Medicine, University of California Los Angeles, Los Angeles, CA USA; 36 Department of Biostatistics, Fielding School of Public Health, University of California Los Angeles, Los Angeles, CA USA; 37 VA Palo Alto Healthcare System, Palo Alto, CA USA; 38 Stanford Cardiovascular Institute, Stanford University, Stanford, CA USA; 39 Cardiovascular Health Research Unit, Departments of Epidemiology, Medicine, and Health Services, University of Washington, Seattle, WA USA; 40 Kaiser Permanente Washington Health Research Institute, Seattle, WA USA; 41 Cardiovascular Health Research Unit, Division of Cardiology, University of Washington, Seattle, WA USA; 42 Stanford Diabetes Research Center, Stanford University, Stanford, CA USA; 43 Department of Biostatistics and Epidemiology, MRC Centre for Environment and Health, School of Public Health, Imperial College, London, UK; 44 Department of Preventive Medicine, Feinberg School of Medicine, Northwestern University, Chicago, IL USA; 45 Department of Medicine, University of North Carolina, Chapel Hill, NC USA; 46 Department of Environmental Health Sciences, Harvard T.H. Chan School of Public Health, Boston, MA USA; 47 Department of Environmental Health Sciences, Columbia University Mailman School of Public Health, New York, NY USA; 48 Framingham Heart Study, Framingham, MA USA

**Keywords:** Genetics, Epigenetics, Epigenomics, Epigenomics, Dyslipidaemias

Correction to: *Nature Communications* 10.1038/s41467-021-23899-y, published online 28 June 2021.

The original version of this Article contained an error in the spelling of the author Min-A Jhun which was incorrectly given as Mina-A Jhun. This has now been corrected in both the PDF and HTML versions of the Article.

