# Supplementary material for: A multi-ethnic epigenome-wide association study of leukocyte DNA methylation and blood lipids
Source: Nat Commun. 2021 Jun 28;12:3987. doi: 10.1038/s41467-021-23899-y (PMC8238961; doi:10.1038/s41467-021-23899-y)
Supplement: Supplementary file 1 — Supplementary Information [file 41467_2021_23899_MOESM1_ESM.pdf]

Supplementary Information

A multi-ethnic epigenome-wide association study of leukocyte DNA methylation and blood lipids

Min A Jhun et al.

Supplementary Methods

Participated study populations (in alphabetical order)

The Amish complex disease research studies (Amish)

The Old Order Amish (OOA) subjects included in this study were participants of several studies of cardiovascular health in relatively healthy volunteers from the OOA community of Lancaster County, PA and their family members. The studies were carried out at the University of Maryland as part of the Amish Complex Disease Research Program (ACDRP) (http://medschool.umaryland.edu/endocrinology/amish/research-program.asp). The OOA population of Lancaster County, PA immigrated to the Colonies from Western Europe in the early 1700's. There are now over 30,000 OOA individuals in the Lancaster area, nearly all of whom can trace their ancestry back 12-14 generations to approximately 750 founders. Investigators at the University of Maryland, School of Medicine have been studying the genetic determinants of cardiometabolic health in this population since 1993. To date, over 7,000 Amish adults have participated in one or more of our studies. The subjects on whom the methylation chip was used were participants of the Heredity and Phenotype Interaction (HAPI) heart study [1], the Pharmacogenomics of Anti-Platelet Intervention (PAPI) study [2], or the Amish Family Diabetes Study (AFDS) (Hsueh WC, Diabetes 2007). These studies collected large numbers of variables including demographic and anthropometric information, medical history, clinical characteristics, lifestyle factors, and study specific variables, as well as blood and urine samples. All study protocols were approved by the institutional review board at the University of Maryland and participating institutions. Informed consent was obtained from each of the study participants.

The Atherosclerosis Risk in Communities (ARIC)

The Atherosclerosis Risk in Communities (ARIC) study is a prospective cohort study of cardiovascular disease risk in 15,792 men and women from four US communities (Forsyth County, North Carolina; Jackson, Mississippi; suburban Minneapolis, Minnesota; and Washington County, Maryland) [3]. In Jackson, only African Americans were enrolled. In the other three communities, samples reflect the demographic composition of the community. 7,082 men and 8,710 women aged 45–64 years were recruited between 1987 and 1989. Written informed consent was obtained and the study protocol was approved by the institutional review board of each

participating university. Participants underwent a baseline clinical examination (Visit 1) and four subsequent follow-up clinical exams (Visits 2–5). DNA sample, lipids and covariate data were from the same study visit. In the current analyses, all data come from Visit 2 or 3 and DNA methylation data are available for African Americans only. Thus, only African Americans were included in the analysis. The ARIC study is approved by the IRBs of the University of Mississippi Medical Center, Wake Forest University Health Sciences, University of Minnesota , and John Hopkins University.

Bogalusa Heart Study (BHS)

The Bogalusa Heart Study is a biracial (black-white) community-based long-term epidemiologic investigation of the early natural history of cardiovascular disease beginning in childhood. Between 1973 and 2010, nine cross-sectional surveys of black and white children aged 4-18 years and eleven cross-sectional surveys of adults, aged 19-58 years, who had been previously examined as children, were conducted in Bogalusa, Louisiana. By linking these 20 surveys, 12,164 children have been examined, with 38,058 serial observations. Among these participants, there are 3,816 adults who had been examined in previous children surveys.
A total number of 958 subjects (676 whites and 282 blacks, age range=28.4-50.6 years) were examined during 2006-2010 for cardiovascular risk factors and blood collection. Study visits were made in the morning after an overnight fast of 12 hours. Demographics, medical history, cardiovascular risk factors, lifestyles, and blood samples were collected. Genome-wide DNA methylation profile was measured by Infinium Human-Methylation450K BeadChip (Illumina, San Diego, CA, USA). All subjects in this study gave informed consent at each examination. Study protocols were approved by the Institutional Review Board of the Tulane University Health Sciences Center.

Cardiovascular Health Study (CHS)

The CHS is a population-based cohort study of risk factors for coronary heart disease and stroke in adults ≥65 years conducted across four field centers [4]. The original predominantly European ancestry cohort of 5,201 persons was recruited in 1989-1990 from random samples of the Medicare eligibility lists; subsequently, an

additional predominantly African-American cohort of 687 persons was enrolled in 1992-1993 for a total sample of 5,888. DNA methylation was measured on 200 European ancestry and 200 African-American ancestry participants. The samples were randomly selected among participants with available DNA at study year 5. The European ancestry participants additionally had no baseline history of coronary heart disease, congestive heart failure, peripheral vascular disease, valvular heart disease, stroke or transient ischemic attack. CHS was approved by institutional review committees at each field center and individuals in the present analysis had available DNA and gave informed consent including consent to use of genetic information for the study of cardiovascular disease. The CHS study is approved by the IRBs at Wake Forest University Health Sciences, University of California, Davis, John Hopkins University, and University of Pittsburgh, and University of Washington (UW 37714-EG).

Framingham Heart Study (FHS)

The Framingham Heart Study Offspring cohort (FHS-Offspring) was initially recruited in 1971 and included 5,124 offspring of the FHS Original cohort. From 2002 to 2005, the adult children (third generation cohort, N=4,095) of the offspring cohort participants were recruited and examined (FHS-3rd Gen). Detailed descriptions of cohorts have been published [5,6]. A total of 2,836 FHS-Offspring participants who attended the eighth exam cycle from 2005-2008 were included in the DNA methylation meta-analysis study. All participants provided written consent for genetic research. The Framingham Heart Study Offspring Cohort examination 8 was approved by the Boston University Medical Center Institutional Review Board (#H-22762) and the DNA methylation subproject by the NHLBI'S IRB (#11-H-N127).

Genetic Epidemiology Network of Arteriopathy (GENOA)

The Genetic Epidemiology Network of Arteiopathy (GENOA) study is a community-based study of hypertensive sibships that was designed to investigate the genetics of hypertension and target organ damage in African Americans from Jackson, Mississippi and non-Hispanic whites from Rochester, Minnesota (Daniels, 2004). In the initial phase of the GENOA study (Phase I: 1996-2001), all members of sibships containing ≥ 2

individuals with essential hypertension clinically diagnosed before age 60 were invited to participate, including both hypertensive and normotensive siblings. Exclusion criteria of the GENOA study were secondary hypertension, alcoholism or drug abuse, pregnancy, insulin-dependent diabetes mellitus, or active malignancy. Eighty percent of African Americans (1,482 subjects) and 75% of non-Hispanic whites (1,213 subjects) from the initial study population returned for the second examination (Phase II: 2001-2005). Study visits were made in the morning after an overnight fast of ten hours. Demographic information, medical history, clinical characteristics, lifestyle factors, and blood samples were collected in each phase. DNA methylation levels were measured only in African Americans participants. Thus, only African Americans were included in the current analysis. Written informed consent was obtained from all subjects and approval was granted by participating institutional review boards. The GENOA study was approved at the IRBs of the University of Michigan, Mayo Clinic, and University of Mississippi Medical Center.

Genetics of Lipid Lowering Drugs and Diet Network (GOLDN)

The Genetics of Lipid Lowering Drugs and Diet Network (GOLDN) is a family intervention study in the PROGENI (PROgram for GENetic Interaction) Network, which investigated genetic determinants of response to two short term interventions including a high-fat meal challenge followed by daily fenofibrate treatment for three weeks. Caucasian individuals from three-generation families were recruited from two Family Heart study field centers (Minneapolis, MN and Salt Lake City, UT). Volunteers were asked to discontinue the use of lipid-lowering agents (pharmaceuticals or nutraceuticals) for at least 4 weeks, and not drink alcohol for at least 24 hours before study visits. A total of 714 individuals met all eligibility requirements and contributed epigenetic data to this project. After signing consent forms, participants underwent a screening visit (visit 0). This screen included a fasting blood draw, and DNA for all participants. If at least two members in a sibship met study criteria including a level of fasting TG of less than 1,500mg/dL, all willing family members were asked to participate in clinical interventions. On the first study visit participants underwent dietary fat challenge meals with 83% of calories from fat and 700 calories per m2 of body surface area. The fasting lipid measures from this visit (ie before the high-fat meal challenge) were used as the variables of interest for this analysis.

Information on demographic, lifestyle, medical history, current medication use, and family history were collected by questionnaires adapted from the Family Heart study. Ancestry was measured via principal component analysis from existing genome-wide association study (GWAs) data. The GOLDN study protocol was approved by IRBs at the University of Minnesota, University of Utah, Tufts University/New England Medical Center, and University of Alabama at Birmingham; all research procedures heeded the principles of the Declaration of Helsinki and all participants provided written informed consent

The Hypertension Genetic Epidemiology Network (HyperGEN)

The Hypertension Genetic Epidemiology Network (HyperGEN) is a family study in the Family Blood Pressure Program which utilized a sib-pair design where each sibling had hypertension onset before age 60 to identify genetic contributors to hypertension [7]. The study was expanded to other siblings of the original pair and their offspring in a second phase [7]. Exclusion criteria included secondary hypertension, and type 1 diabetes mellitus (insulin therapy before age 21 years), and renal disease [8]. African Americans were recruited at Forsyth County, NC and Birmingham, AL centers from 1995 to 2000 [7]. A total of 604 African American participants met all eligibility requirements and contributed to epigenetic data to this project. Participants were asked to fast for 12 hours before withdrawing blood. HDL-C was measured after precipitation of non–HDL-C with magnesium/dextran [9]. TG were measured by using glycerol-blanked TG reagent on a centrifugal analyzer [9]. LDL-C was measured by using standard methods or by ultracentrifugation for subjects with TG levels >400 mg/dL [9]. Total cholesterol was measured by using a commercial cholesterol oxidase method [9]. Ancestry was measured via principal component analysis from existing exome chip data for LDL-C and TG, and genome-wide association study (GWAs) data for HDL-C. All HyperGEN participants provided informed consent for use of samples and data for subsequent analyses.

Cooperative health research in the Region of Augsburg (KORA)

The KORA study (Cooperative health research in the Region of Augsburg) is an independent population-based cohort from the region of Augsburg, Southern Germany operated by the Helmholtz Zentrum München to

examine the links between health, disease and the living conditions of the population. Participants were recruited from the general population of Augsburg and the surrounding districts. The S4 survey (examination 1999-2001) consisted of standardized interviews, physical examinations and blood sampling. Only individuals between the ages of 25 and 74 were selected. For the CHARGE lipids study, participants of the KORA F4 cohort (examination 2006-2008), a seven-year follow-up study of the KORA S4 cohort, were used. Again, the F4 survey consisted of standardized interviews, physical examinations and blood sampling. All participants gave written informed consent and this study was approved by the local ethics committee (Bayerische Landesärztekammer). Of the 1799 individuals for whom methylation data were available, 72 failed to pass quality control criteria and 8 were not in a fasting state at the time of DNA extraction. Of those remaining, a total of 1655 individuals also had genetic (SNP) data available, but four lacked the necessary covariate data for the models, leaving a total of 1651 individuals available for the study.   The KORA study has been approved by the Bayerische Landesärztekammer (IRB: 00001087).

The Normative Aging Study (NAS)

The ongoing longitudinal US Department of Veterans Affairs (VA) Normative Aging Study (NAS) was established in 1963 and included men, 21-80 years old and free of known chronic medical conditions at entry [10]. Subsequently participants were invited to medical examinations every three to five years. At each visit, men provided information on medical history, lifestyle, and demographic factors, and underwent physical examinations and laboratory tests. DNA samples were collected from active participants between 1999-20131. In total, 674 individuals (656 white, 12 black, 5 Hispanic white, 1 Hispanic black) with single observation each are included in this analysis. Participants have provided written informed consent at each visit. The NAS study was approved by the Institutional Review Boards (IRBs) of the participating institutions.   The NAS study was approved by the Institutional Review Board of the Harvard T.H. Chan School of Public Health.

The Prospective Investigation of Vascularity of Uppsala Elders Study (PIVUS)

PIVUS is a prospective community-based cohort of participants from Uppsala, Sweden. All men and women at age 70 living in Uppsala in 2001 were invited to participate. The 1,016 participants (50% women) have been extensively phenotyped, as described previously [11]

, and on the Internet (www.medsci.uu.se/pivus/). All individuals have undergone a detailed medical examination including a detailed questionnaire on lifestyle and socioeconomic factors, fasting blood sampling, blood pressure measurement and anthropometric measurements at baseline. Lipid traits were measured at Uppsala University Hospital using routine medical chemistry methods following fasting. LDL-C was calculated by the Friedewald equation [12]. The participants have been re-examined at ages 75 and 80, and their morbidity and mortality has been followed via national registers and journal review. All participants have provided written informed consent, and the studies have been reviewed and approved by the Ethics Committee at Uppsala University.

Rotterdam Study (RS)

The Rotterdam Study is a prospective population-based cohort study in a well-defined area of Rotterdam, the Netherlands. For the current analysis we used data from individuals aged 45 years and older that participated in the third cohort of the Rotterdam Study. In the first visit of the third cohort, 3934 participants were examined between February 2006 and December 2008. Whole blood DNA methylation was quantified in a random subset of 731 individuals. During the research center visit, fasting blood samples were collected and anthropometric measures were obtained. Smoking behavior (current, former and never) was assessed during home interview by trained research assistants. The Rotterdam Study has been approved by the Medical Ethics Committee of the Erasmus MC and by the Ministry of Health, Welfare and Sport of the Netherlands, implementing the “Wet Bevolkingsonderzoek: ERGO (Population Studies Act: Rotterdam Study)”. All participants provided written informed consent to participate in the study and to obtain information from their treating physicians.

The UK Adult Twin Registry (TwinsUK)

The TwinsUK cohort was established in 1992 to recruit monozygotic and dyzygotic twins same-sex adult twins [13]. The majority of participants are healthy female subjects of European ancestry (age range from 16 to 98 years old). There are more than 13,000 twin participants from all regions across the United Kingdom and many have multiple visits over the years. The TwinsUK cohort has been used in many epidemiological studies, and is representative of the general UK population for a wide range of diseases and traits. Members of TwinsUK have been shown to have similar disease and lifestyle characteristic to the general population. Ethical approval was granted by the National Research Ethics Service London-Westminster, the St Thomas' Hospital Research Ethics Committee (EC04/015 and 07/H0802/84). All research participants have signed informed consent prior to taking part in the research study.

The Women's Health Initiative (WHI-BA23)

Women were selected from one of two WHI large sub cohorts that had previously undergone genome wide genotyping as well as profiling for 7 cardiovascular disease related biomarkers including total cholesterol, HDL, LDL, triglycerides, CRP, creatinine, insulin, and glucose through 2 core WHI ancillary studies. The first cohort is the WHI SNP Health Association Resource (SHARe) cohort of minorities that includes >8000 African American (AA) women and >3500 Hispanic women. These women were genotyped through WHI core study M5-SHARe (www.whi.org/researchers/data/WHIStudies/StudySites/M5) and underwent biomarker profile through WHI Core study W54-SHARe (...data/WHIStudies/StudySites/W54). The second cohort consists of a combination of European Americans (EA) from the two Hormonal Therapy (HT) trials selected for GWAS and biomarkers in core studies W58 (.../data/WHIStudies/StudySites/W58) and W63 (.../data/WHIStudies/StudySites/W63). From these two cohorts, two sample sets were formed. The first (sample set 1) is a sample set of 637 CHD cases and 631 non-CHD cases as of Sept 30, 2010. The second sample set (sample set 2) is a non-overlapping sample of 432 cases of coronary heart disease and 472 non-cases as of September 17, 2012. All women with measures of inflammation that passed QC were included in this analysis. The WHI-BA23 study had been approved by the IRB of the Stanford University (IRB-34553).

Women's Health Initiative Epigenetic Mechanisms of PM-Mediated CVD (WHI-EMPC)

WHI-EMPC is an ancillary study of epigenetic mechanisms underlying associations between ambient particulate matter (PM) air pollution and cardiovascular disease (CVD) in the Women's Health Initiative clinical trials (CT) cohort. It is funded by the National Institute of Environmental Health Sciences (R01-ES020836). The WHI-EMPC study population is a stratified, random sample of 2,200 WHI CT participants who were examined between 1993 and 2001; had available buffy coat, core analytes, electrocardiograms, and ambient concentrations of PM; but were not taking anti-arrhythmic medications at the time. As such, WHI-EMPC is representative of the larger, multiethnic WHI CT population from which it was sampled: n = 68,132 participants aged 50-79 years who were randomized to hormone therapy, calcium / vitamin D supplementation, and / or dietary modification in 40 U.S. clinical centers at the baseline exam (1993-1998) and re-examined in the fasting state one, three, six, and nine years later [14,15]. The WHI-EPMC study had been approved by the IRB of the University of North Carolina.

Supplementary Figures

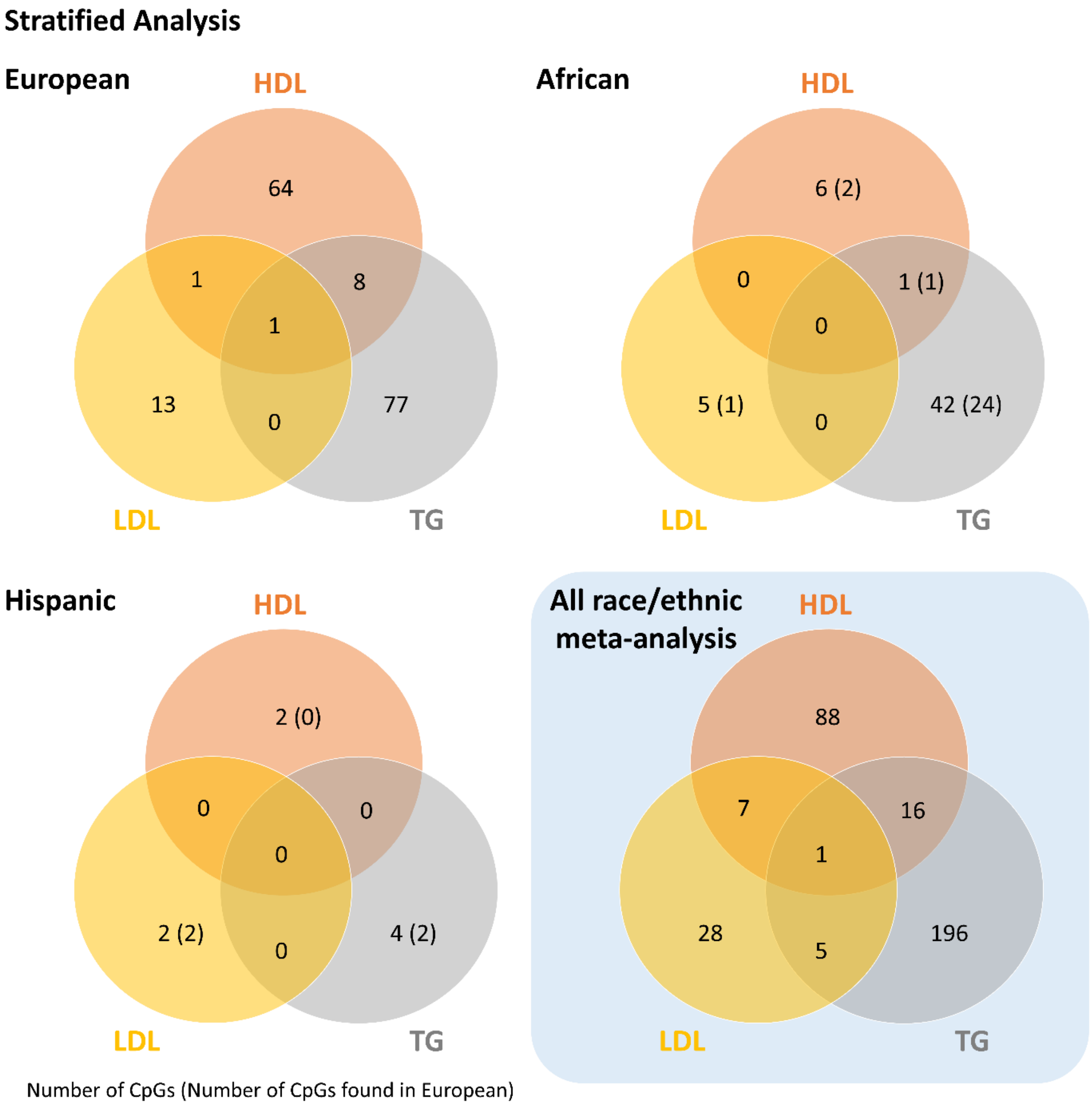

Supplementary Figure 1. Number of significant CpGs identified in the race/ethnic specific (European, African, and Hispanic) stratified meta-analysis and all trans-ethnic meta-analysis (lower right) for high-density lipoprotein (HDL), low-density lipoprotein (LDL), and/or triglycerides (TG) levels after adjusting for BMI and excluding subjects on statins (model 4). Numbers in parentheses indicate the number of CpGs also identified in European ancestry population.

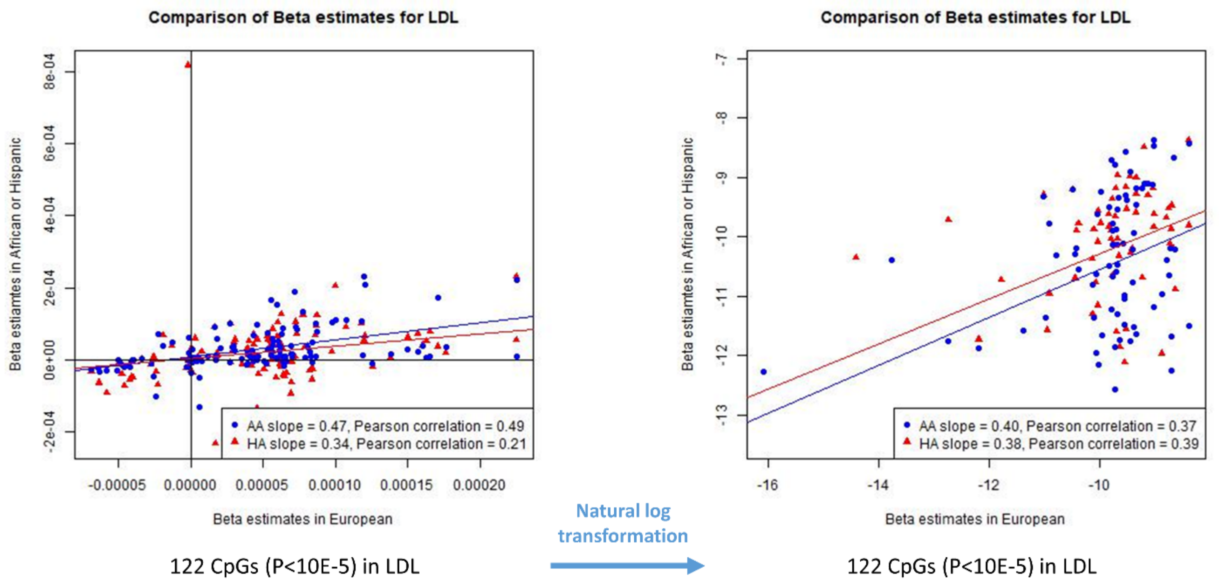

Supplementary Figure 2. Scatter plots and regression lines of beta estimate pairs observed in two racial/ethnic groups for 122 CpG-low density lipoprotein (LDL) associations found to be significant at $P<1.09\times10^{-5}$ in one or more racial/ethnic group. Panels shows plots and regression lines before (left) and after (right) natural log transformation of LDL. In both panels, scatter plots and regression lines are shown for the European and African American (AA) pair of betas (blue circles/lines) as well as the European and Hispanic (HA) pair of betas (red triangles/lines). Numerical regression slope and Pearson correlation coefficients are presented in the bottom right corner of each plot.

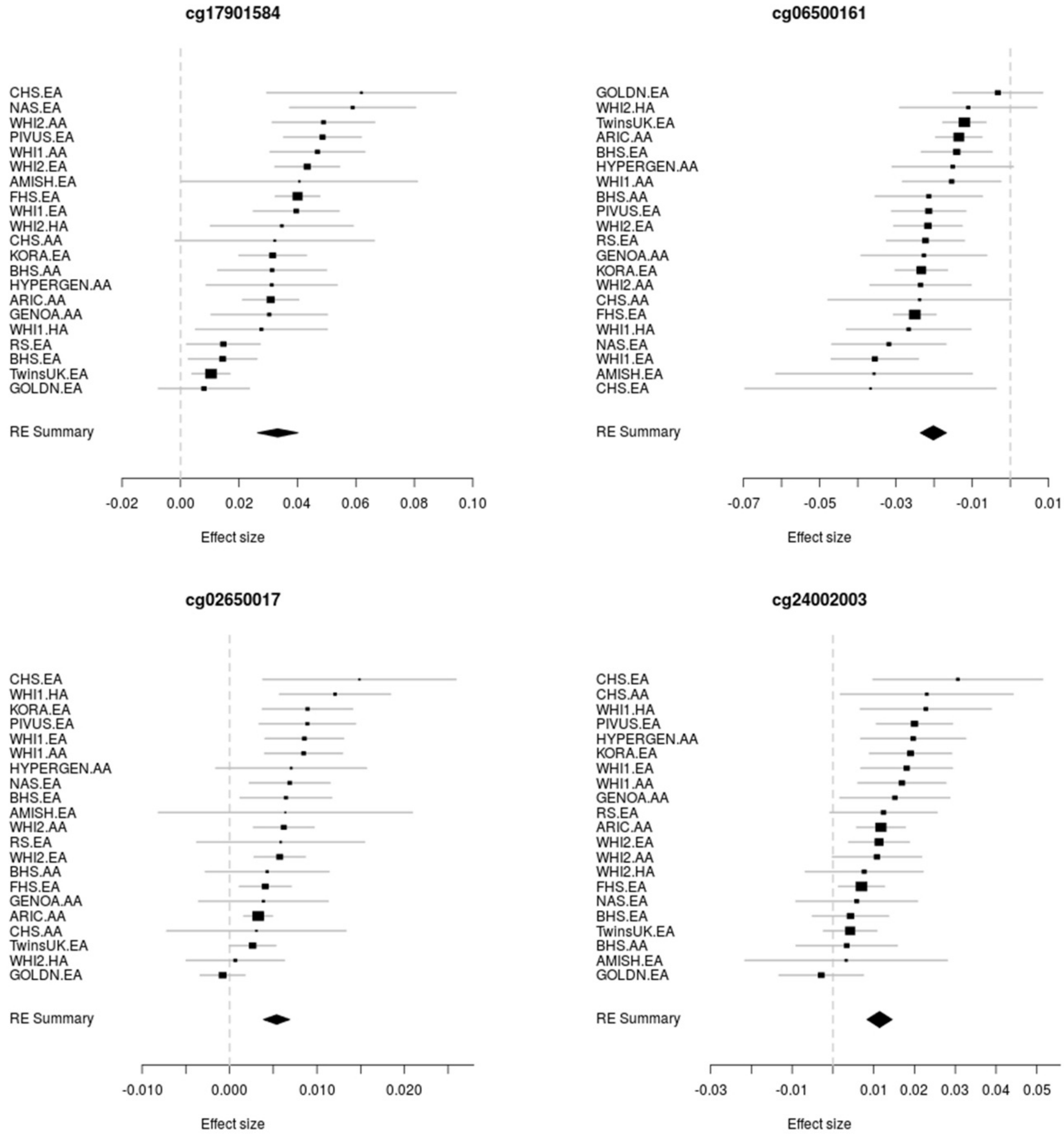

Supplementary Figure 3. Forest plots of the four trans-ethnic CpGs associated with high-density lipoprotein. Cohorts include the Old Order Amish (AMISH), Atherosclerosis Risk in Communities (ARIC), Bogalusa Heart Study (BHS), Cardiovascular Health Study (CHS), Framingham Heart Study (FHS), Genetic Epidemiology Network of Arteriopathy (GENOA), Genetics of Lipid Lowering Drugs and Diet Network (GOLDN), Hypertension Genetic Epidemiology Network (HyperGEN), Cooperative health research in the Region of Augsburg (KORA), Normative Aging Study (NAS), Prospective Investigation of Vascularity of Uppsala Elders Study (PIVUS), Rotterdam Study (RS), UK Adult Twin Registry (TwinsUK), Women's Health Initiative Broad Agency Announcement 23 (WHI1), and the Women's Health Initiative Epigenetic Mechanisms of PM-Mediated CVD (WHI2) cohorts. .EA: European ancestry subgroup; .AA: African ancestry subgroup; .HA: Hispanic ancestry subgroup; $N_{.EA}$ = 11,114, $N_{.AA}$ = 4,452, $N_{.HA}$ = 699; The statistic is a likelihood ratio test (LRT) which asymptotically follows an equal mixture of 1 degree of freedom (df) $\chi 2$ distribution and 2 df $\chi 2$ distribution; A p value was considered significant in a specific racial/ethnic group if it was < 1.09 × 10-7 (Bonferroni correction for the number of CpG probes tested). Mid-point of the box represents the point effect estimate for each study. The area of the box represents the weight given to the study (proportional to sample size). The horizontal line through each box represents the 95% confidence interval for the point effect estimate. The diamond shows the confidence interval for the overall effect estimate.

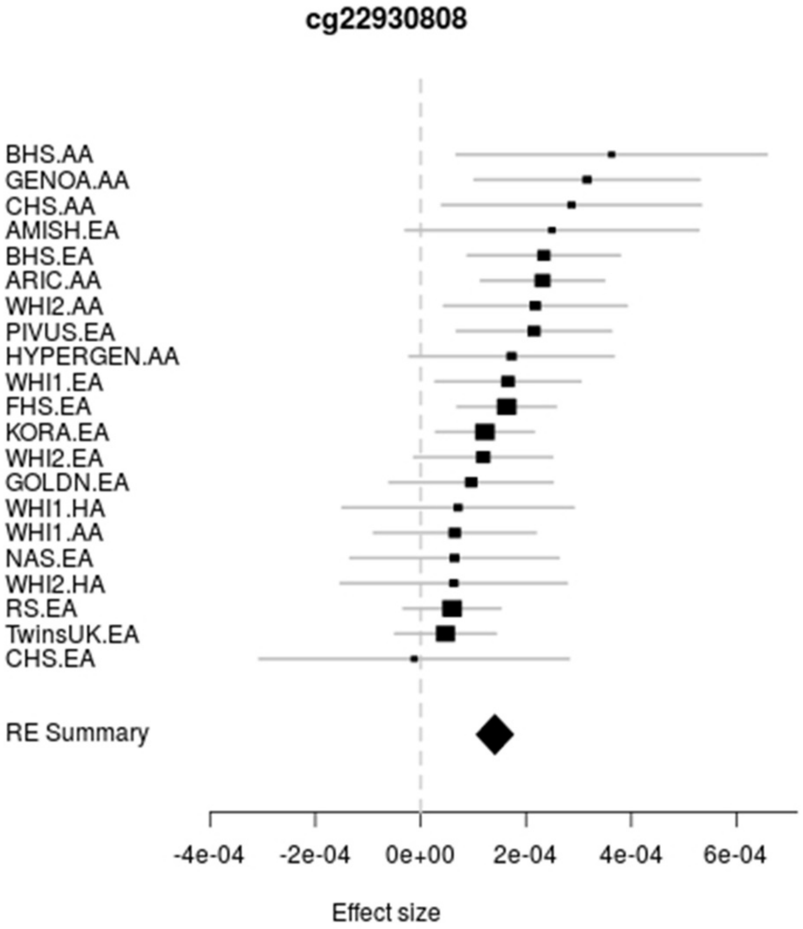

Supplementary Figure 4. Forest plots of the trans-ethnic CpGs associated with low-density lipoprotein. Cohorts include the Old Order Amish (AMISH), Atherosclerosis Risk in Communities (ARIC), Bogalusa Heart Study (BHS), Cardiovascular Health Study (CHS), Framingham Heart Study (FHS), Genetic Epidemiology Network of Arteriopathy (GENOA), Genetics of Lipid Lowering Drugs and Diet Network (GOLDN), Hypertension Genetic Epidemiology Network (HyperGEN), Cooperative health research in the Region of Augsburg (KORA), Normative Aging Study (NAS), Prospective Investigation of Vascularity of Uppsala Elders Study (PIVUS), Rotterdam Study (RS), UK Adult Twin Registry (TwinsUK), Women's Health Initiative Broad Agency Announcement 23 (WHI1), and the Women's Health Initiative Epigenetic Mechanisms of PM-Mediated CVD (WHI2) cohorts. .EA: European ancestry subgroup; .AA: African ancestry subgroup; .HA: Hispanic ancestry subgroup; $N_{.EA}$ = 11,114, $N_{.AA}$ = 4,452, $N_{.HA}$ = 699; The statistic is a likelihood ratio test (LRT) which asymptotically follows an equal mixture of 1 degree of freedom (df) $\chi 2$ distribution and 2 df $\chi 2$ distribution; A p value was considered significant in a specific racial/ethnic group if it was < 1.09 × 10-7 (Bonferroni correction for the number of CpG probes tested). Mid-point of the box represents the point effect estimate for each study. The area of the box represents the weight given to the study (proportional to sample size). The horizontal line through each box represents the 95% confidence interval for the point effect estimate. The diamond shows the confidence interval for the overall effect estimate.

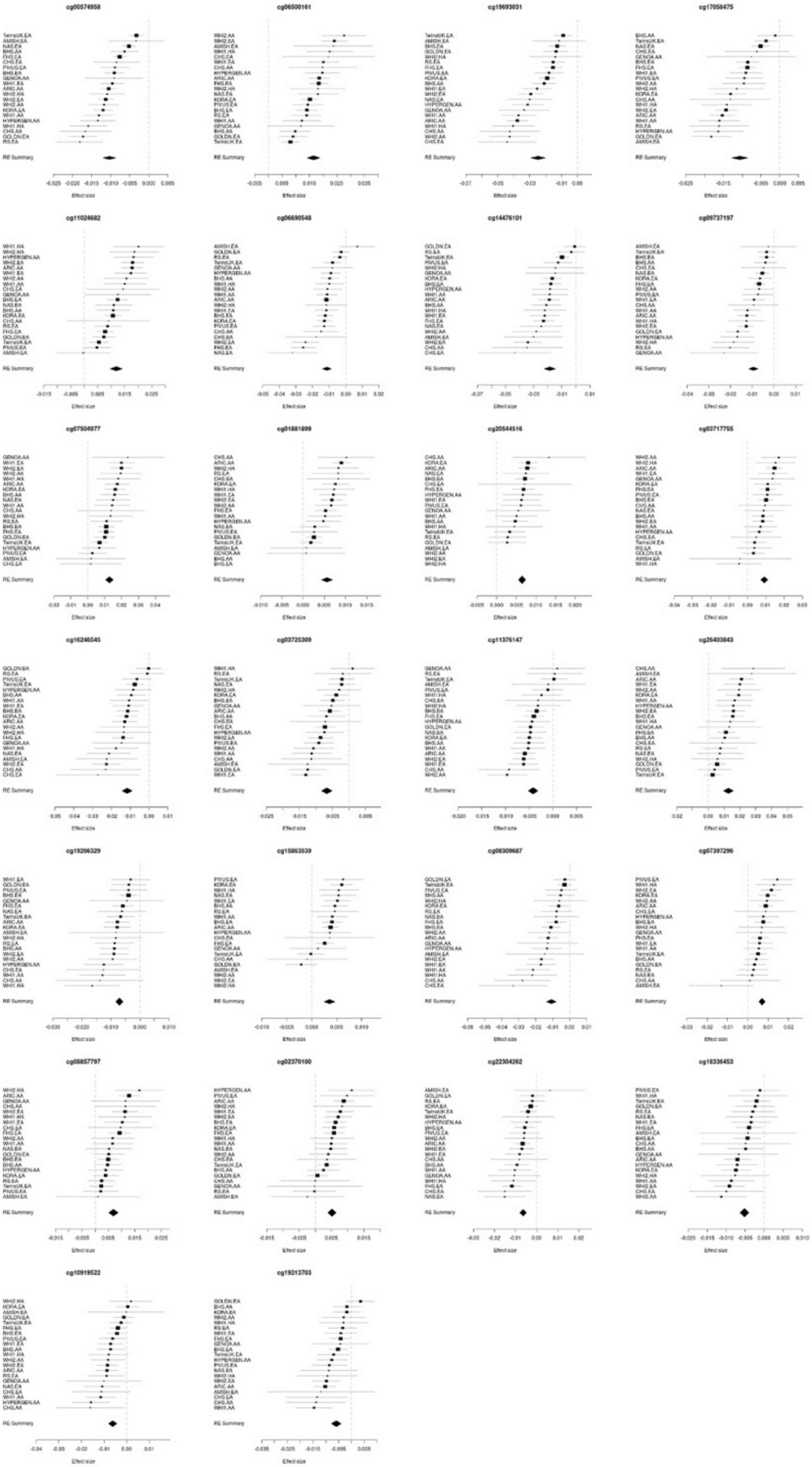
cg00574958
cg06500161
cg19693031
cg17058475
cg11024682
cg06690548
cg14476101
cg09737197
cg07504977
cg01881899
cg20544516
cg03717755
cg16246545
cg03725309
cg11376147
cg26403843
cg19266329
cg15863539
cg08309687
cg07397296
cg08857797
cg02370100
cg22304262
cg18336453
cg10919522
cg19213703
RE Summary
Effect size

Supplementary Figure 5. Forest plots of the 26 trans-ethnic CpGs associated with triglycerides. Cohorts include the Old Order Amish (AMISH), Atherosclerosis Risk in Communities (ARIC), Bogalusa Heart Study (BHS), Cardiovascular Health Study (CHS), Framingham Heart Study (FHS), Genetic Epidemiology Network of Arteriopathy (GENOA), Genetics of Lipid Lowering Drugs and Diet Network (GOLDN), Hypertension Genetic Epidemiology Network (HyperGEN), Cooperative health research in the Region of Augsburg (KORA), Normative Aging Study (NAS), Prospective Investigation of Vascularity of Uppsala Elders Study (PIVUS), Rotterdam Study (RS), UK Adult Twin Registry (TwinsUK), Women's Health Initiative Broad Agency Announcement 23 (WHI1), and the Women's Health Initiative Epigenetic Mechanisms of PM-Mediated CVD (WHI2) cohorts. EA: European ancestry subgroup; .AA: African ancestry subgroup; .HA: Hispanic ancestry subgroup; $N_{.EA}$ = 11,114, $N_{.AA}$ = 4,452, $N_{.HA}$ = 699; The statistic is a likelihood ratio test (LRT) which asymptotically follows an equal mixture of 1 degree of freedom (df) $\chi 2$ distribution and 2 df $\chi 2$ distribution; A p value was considered significant in a specific racial/ethnic group if it was < 1.09 × 10-7 (Bonferroni correction for the number of CpG probes tested). Mid-point of the box represents the point effect estimate for each study. The area of the box represents the weight given to the study (proportional to sample size). The horizontal line through each box represents the 95% confidence interval for the point effect estimate. The diamond shows the confidence interval for the overall effect estimate.

Supplementary Tables

Supplementary Table 1. Lamda (**λ**) for mean effect and heterogeneity for each epigenome wide association study model before and after genomic control (GC)

EWAS MODEL	**λ** for mean effect	**λ** for heterogeneity	**λ** for mean effect after GC	**λ** for heterogeneity after GC
HDL.MODEL1.AA.GC	1.245963	1.389998	1	1
HDL.MODEL1.ALL_wGOLDN.GC	1.919261	1.357792	1	1
HDL.MODEL1.EA_wGOLDN.GC	1.856773	1.158513	1	1
HDL.MODEL1.HA.GC	1.132357	0.480564	1	1
HDL.MODEL2.AA.GC	1.177791	1.264493	1	1
HDL.MODEL2.ALL_wGOLDN.GC	1.599381	1.131483	1	1
HDL.MODEL2.EA_wGOLDN.GC	1.514254	0.98327	1	1
HDL.MODEL2.HA.GC	1.118549	0.470066	1	1
HDL.MODEL3.AA.GC	1.268461	1.546222	1	1
HDL.MODEL3.ALL_wGOLDN.GC	1.843344	1.298639	1	1
HDL.MODEL3.EA_wGOLDN.GC	1.711385	1.057492	1	1
HDL.MODEL3.HA.GC	1.050254	0.471509	1	1
HDL.MODEL4.AA.GC	1.207928	1.375914	1	1
HDL.MODEL4.ALL_wGOLDN.GC	1.570446	1.095986	1	1
HDL.MODEL4.EA_wGOLDN.GC	1.415135	0.919724	1	1
HDL.MODEL4.HA.GC	1.049831	0.430773	1	1
LDL.MODEL1.AA.GC	1.000749	0.891525	1	1
LDL.MODEL1.ALL_wGOLDN.GC	1.210873	0.981682	1	1
LDL.MODEL1.EA_wGOLDN.GC	1.237366	0.899142	1	1
LDL.MODEL1.HA.GC	1.092019	0.541441	1	1
LDL.MODEL2.AA.GC	0.991213	0.845909	1	1
LDL.MODEL2.ALL_wGOLDN.GC	1.229201	0.976123	1	1
LDL.MODEL2.EA_wGOLDN.GC	1.248973	0.913112	1	1
LDL.MODEL2.HA.GC	1.100346	0.584253	1	1
LDL.MODEL3.AA.GC	1.003006	0.972559	1	1
LDL.MODEL3.ALL_wGOLDN.GC	1.231532	1.105697	1	1
LDL.MODEL3.EA_wGOLDN.GC	1.309409	1.028566	1	1
LDL.MODEL3.HA.GC	1.073513	0.500554	1	1
LDL.MODEL4.AA.GC	0.993391	0.951705	1	1
LDL.MODEL4.ALL_wGOLDN.GC	1.253716	1.103539	1	1
LDL.MODEL4.EA_wGOLDN.GC	1.340334	1.036487	1	1
LDL.MODEL4.HA.GC	1.075197	0.540851	1	1
TG.MODEL1.AA.GC	1.164986	1.011341	1	1
TG.MODEL1.ALL_wGOLDN.GC	1.347646	1.084994	1	1
TG.MODEL1.EA_wGOLDN.GC	1.244159	1.033869	1	1
TG.MODEL1.HA.GC	1.068191	0.58933	1	1
TG.MODEL2.AA.GC	1.119791	0.916542	1	1
TG.MODEL2.ALL_wGOLDN.GC	1.201488	0.961739	1	1
TG.MODEL2.EA_wGOLDN.GC	1.117851	0.930156	1	1
TG.MODEL2.HA.GC	1.076921	0.55404	1	1
TG.MODEL3.AA.GC	1.193242	1.014136	1	1
TG.MODEL3.ALL_wGOLDN.GC	1.289871	0.9822	1	1
TG.MODEL3.EA_wGOLDN.GC	n/a	n/a		
TG.MODEL3.HA.GC	1.040557	0.471006	1	1
TG.MODEL4.AA.GC	n/a	n/a		
TG.MODEL4.ALL_wGOLDN.GC	1.172445	0.909941	1	1
TG.MODLE4.EA_wGOLDN.GC	n/a	n/a		
TG.MODEL4.HA.GC	1.028641	0.470758	1	1

HDL: high-density lipoprotein; LDL: low-density lipoprotein; TG: triglycerides; EA: European ancestry; AA: African ancestry; HA: Hispanic ancestry; **λ**: lambda; GC: genomic control

Supplementary Table 2. Characteristics of study samples in prior Epigenome-wide Association Studies related to cardiometabolic traits used to assess overlap with findings for lipid traits in this study

Trait	Year	Journal	Race/ethnicity	Sample size
BMI	2017	Nature	European, Indian Asian	10261 = 5387 (Discovery) + 4874 (Replication)
Hepatic fat	2019	Diabetes	European, African, Hispanic	4525 = 3400 (EA) + 401 (HA) + 724 (AA)
Fasting insulin or HOMA-IR	2014	Diabetes	European	837 = 544 (Discovery) + 293 (replication)
Incident T2D	2015	Lancet Diabetes Endocrinol	Indian Asian, European	20601 = 13535 (Indian Asian) + 7066 (European)
eGFR	2017	Ncomm	European, African	4859 = 2264 (AA) + 2595 (EA)
Blood pressure	2017	AJHG	European, African, Hispanic	17010
CRP	2016	Genome Biology	European, African	12974 = 8863 (European discovery) + 4111 (African replication)
Smoking	2016	Circ Cardiovasc Genet	European, African, Hispanic	15907

EA: European ancestry; AA: African ancestry; HA: Hispanic ancestry

Supplementary Table 3. Genome characteristics of 7 CpGs associated with lipid trait in more than one racial/ethnic group also found to be associated with the expression of the respective gene mapped gene in the Framingham Heart Study

CpG	In previous lipid EWAS?	Genome Build	CHR	MAPINFO	Gene	Group	UCSC CpG Islands Name	Relation to UCSC CpG Island	DMR	Enhancer	DHS	Transcript (Hg19)	Beta	P
cg14476101	Y	37	1	120255992	PHGDH	Body	chr1:120254844-120255499	S_Shore	RDMR			1:120202441-120286788	-0.4	4.09E-09
cg22930808	N	37	3	122281881	PARP9	5'UTR	chr3:122283002-122283594	N_Shore				3:122398047-122449684	-0.5	5.58E-10
cg22930808	N	37	3	122281881	PARP9	5'UTR	chr3:122283002-122283594	N_Shore				3:122246779-122283503	-0.4	3.27E-06
cg06690548	Y	37	4	139162808	SLC7A11	Body						4:139060406-139242140	-0.7	3.34E-12
cg09737197	Y	37	11	68607675	CPT1A	5'UTR	chr11:68608155-68609419	N_Shore				11:68522090-68610512	-1.8	2.67E-10
cg17058475	Y	37	11	68607737	CPT1A	5'UTR	chr11:68608155-68609419	N_Shore				11:68522090-68610512	-2.7	1.18E-11
cg07397296	N	37	21	43655316	ABCG1	Body	chr21:43654846-43655465	Island			TRUE	21:43619809-43726380	-0.6	6.26E-08
cg06500161	Y	37	21	43656587	ABCG1	Body	chr21:43654846-43655465	S_Shore		TRUE		21:43619809-43726380	-1.9	4.44E-53

DMR: differentially methylated region; RDMR: reprogrammed differentially methylated region; DHS: DNAse I hypersensitive site. A linear mixed effects model was used to assess association between residuals of DNA methylation and residuals of gene expression levels in the Framingham Heart Study (N = 4,278 participants). The statistic is a two-sided likelihood ratio test (LRT) which asymptotically follows an equal mixture of 1 degree of freedom (df) χ^2 distribution and 2 df χ^2 distribution. Significance was set at 0.05/18 CpGs mapped to genes tested (P<0.0026) .

Supplementary Table 4. Genetic instruments (rs IDs and position build 37) used for Mendelian Randomization study of lipids to CpG methylation associated with lipids

High density lipoprotein (HDL)					Low density lipoprotein (LDL)					Triglycerides				
SNP	Chr	Position	raising allele	Other allele	SNP	Chr	Position	raising allele	Other allele	SNP	Chr	Position	raising allele	Other allele
rs4660293	1	40028180	A	G	rs12027135	1	25775733	T	A	rs2131925	1	63025942	T	G
rs1689800	1	182168885	A	G	rs2479409	1	55504650	G	A	rs4846914	1	2.3E+08	G	A
rs4846914	1	230295691	A	G	rs2131925	1	63025942	T	G	rs1042034	2	21225281	T	C
rs1042034	2	21225281	C	T	rs629301	1	109818306	T	G	rs1260326	2	27730940	T	C
rs12328675	2	165540800	C	T	rs2642442	1	220973563	T	C	rs10195252	2	1.66E+08	T	C
rs2972146	2	227100698	G	T	rs514230	1	234858597	T	A	rs2972146	2	2.27E+08	T	G
rs13107325	4	103188709	C	T	rs1367117	2	21263900	A	G	rs645040	3	1.36E+08	T	G
rs6450176	5	53298025	G	A	rs4299376	2	44072576	G	T	rs442177	4	88030261	T	G
rs2814944	6	34552797	G	A	rs12916	5	74656539	C	T	rs9686661	5	55861786	T	C
rs605066	6	139829666	T	C	rs6882076	5	156390297	C	T	rs6882076	5	1.56E+08	C	T
rs17145738	7	72982874	T	C	rs3757354	6	16127407	C	T	rs13238203	7	72129667	C	T
rs4731702	7	130433384	T	C	rs1800562	6	26093141	G	A	rs17145738	7	72982874	C	T
rs9987289	8	9183358	G	A	rs9488822	6	116312893	A	T	rs11776767	8	10683929	C	G
rs12678919	8	19844222	G	A	rs1564348	6	160578860	C	T	rs1495741	8	18272881	G	A
rs2293889	8	116599199	G	T	rs12670798	7	21607352	C	T	rs12678919	8	19844222	A	G
rs2954029	8	126490972	T	A	rs2072183	7	44579180	C	G	rs2954029	8	1.26E+08	A	T
rs581080	9	15305378	C	G	rs9987289	8	9183358	G	A	rs10761731	10	65027610	A	T
rs1883025	9	107664301	C	T	rs2081687	8	59388565	T	C	rs2068888	10	94839642	G	A
rs2923084	11	10388782	A	G	rs2954029	8	126490972	A	T	rs174546	11	61569830	T	C
rs3136441	11	46743247	C	T	rs11136341	8	145043543	G	A	rs964184	11	1.17E+08	G	C
rs174546	11	61569830	C	T	rs2255141	10	113933886	A	G	rs11613352	12	57792580	C	T
rs964184	11	116648917	C	G	rs174546	11	61569830	C	T	rs4765127	12	1.24E+08	G	T
rs7941030	11	122522375	C	T	rs964184	11	116648917	G	C	rs2412710	15	42683787	A	G
rs7134375	12	20473758	A	C	rs11220462	11	126243952	A	G	rs2929282	15	44245931	T	A
rs11613352	12	57792580	T	C	rs11065987	12	112072424	A	G	rs1532085	15	58683366	A	G
rs7134594	12	110000193	T	C	rs1169288	12	121416650	C	A	rs11649653	16	30918487	C	G
rs4759375	12	123796238	T	C	rs8017377	14	24883887	A	G	rs3764261	16	56993324	C	A
rs4765127	12	124460167	T	G	rs3764261	16	56993324	C	A	rs10401969	19	19407718	T	C
rs838880	12	125261593	C	T	rs2000999	16	72108093	A	G	rs439401	19	45414451	C	T
rs1532085	15	58683366	A	G	rs7206971	17	45425115	A	G	rs6065906	20	44554015	C	T
rs2652834	15	63396867	G	A	rs6511720	19	11202306	G	T	rs5756931	22	38546033	T	C
rs3764261	16	56993324	A	C	rs10401969	19	19407718	T	C					
rs16942887	16	67928042	A	G	rs4420638	19	45422946	G	A					
rs2925979	16	81534790	C	T	rs2902940	20	39091487	A	G					
rs11869286	17	37813856	C	G	rs6029526	20	39672618	A	T					
rs4148008	17	66875294	C	G										
rs4129767	17	76403984	A	G										
rs7241918	18	47160953	T	G										
rs12967135	18	57849023	G	A										
rs7255436	19	8433196	A	C										
rs737337	19	11347493	T	C										
rs4420638	19	45422946	A	G										
rs386000	19	54792761	C	G										
rs1800961	20	43042364	C	T										
rs6065906	20	44554015	T	C										
rs181362	22	21932068	C	T										

Chr: chromosome

Supplementary Table 5. Results of Mendelian Randomization study of lipid trait to CpG using genetic risk scores (GRS) of lipid traits as instruments

CpG	beta	se	statistic	p
**HDL->DNA methylation**				
cg17901584	0.029349	0.015689	1.870742	0.061381
cg02650017	-0.00397	0.003902	-1.01729	0.309017
cg06500161	-0.00721	0.011061	-0.65194	0.514439
cg24002003	-0.00493	0.011632	-0.42346	0.671958
**LDL->DNA methylation**				
cg22930808	-4.92E-05	0.000152	-0.32447	0.745581
**TG->DNA methylation**				
cg00574958	-0.0202	0.004391	-4.59952	4.23E-06
cg17058475	-0.02279	0.00652	-3.49604	0.000472
cg09737197	-0.02243	0.00764	-2.93515	0.003334
cg06500161	0.011794	0.006725	1.753597	0.0795
cg06690548	0.012692	0.008354	1.519217	0.128708
cg07504977	0.014971	0.009968	1.501917	0.133119
cg08857797	0.012243	0.008488	1.442417	0.149185
cg19693031	-0.01389	0.010562	-1.31535	0.188392
cg11024682	0.00733	0.006418	1.142012	0.253449
cg22304262	0.007394	0.007473	0.989357	0.322488
cg03725309	0.006016	0.006351	0.947229	0.343522
cg16246545	-0.00976	0.010435	-0.93535	0.349609
cg15863539	-0.00301	0.003819	-0.78887	0.43019
cg07397296	0.005785	0.007804	0.741261	0.458535
cg19213703	-0.00606	0.008544	-0.70957	0.477969
cg18336453	-0.00436	0.006846	-0.63747	0.52382
cg01881899	0.001919	0.003756	0.510787	0.6095
cg19266329	-0.00334	0.008357	-0.39994	0.689203
cg02370100	-0.00202	0.005605	-0.36121	0.717943
cg14476101	-0.00411	0.012165	-0.33746	0.73577
cg03717755	-0.00326	0.010916	-0.2991	0.764863
cg11376147	-0.00098	0.004207	-0.23215	0.816422
cg26403843	0.00197	0.01108	0.177748	0.858921
cg08309687	0.00112	0.010791	0.103794	0.917332
cg10919522	-0.00063	0.007533	-0.08384	0.933182
cg20544516	8.16E-05	0.005361	0.015228	0.987851

HDL: high-density lipoprotein; LDL: low-density lipoprotein; TG: triglycerides. N = 3376 from 4 cohorts participating in the MR component of this study. Study specific coefficients and standard errors for GRS-lipid and GRS-CpG regressions were combined through standard fixed-effects meta-analysis. Causal beta effects shown above were then estimate through the ratio of coefficients (beta). Standard errors of the ration were calculated using the normal approximation method.

Supplementary Table 6. Results of Mendelian Randomization study CpG to triglyceride

CpG	SNP	Method	Estimate	Std	Lower CI	Upper CI	P
cg01881899	rs4148117	IVW	2.221	1.75	-1.209	5.65	0.204
cg02370100	rs13052462, rs225443, rs881395, rs915847	IVW	1.177	0.503	0.191	2.162	0.019
		MR-Egger	-1.46	21.36	-43.326	40.406	0.946
		(intercept)	0.013	0.109	-0.2	0.226	0.902
cg08309687	rs2834309	IVW	-0.011	0.178	-0.36	0.339	0.952
cg14476101	rs1886736, rs3790707	IVW	-0.027	0.195	-0.409	0.356	0.891
cg16246545	rs1886736, rs894079, rs942835	IVW	-0.42	0.251	-0.912	0.072	0.094
		MR-Egger	0.671	1.553	-2.372	3.715	0.665
		(intercept)	-0.018	0.025	-0.066	0.031	0.476
cg22304262	rs8105903	IVW	0.257	0.729	-1.171	1.686	0.724
cg26403843	rs17056626, rs1897565, rs2163775, rs6556407, rs6556408, rs6888950, rs7721001	IVW	-0.201	0.179	-0.552	0.149	0.26
		MR-Egger	0.946	1.412	-1.821	3.713	0.503
		(intercept)	-0.012	0.015	-0.041	0.017	0.413

IVW: inverse variance weighted

Supplementary References

1. Mitchell, B.D. *et al.* The genetic response to short-term interventions affecting cardiovascular function: rationale and design of the Heredity and Phenotype Intervention (HAPI) Heart Study. *Am Heart J* **155**, 823-8 (2008).
2. Shuldiner, A.R. *et al.* Association of cytochrome P450 2C19 genotype with the antiplatelet effect and clinical efficacy of clopidogrel therapy. *JAMA* **302**, 849-57 (2009).
3. The Atherosclerosis Risk in Communities (ARIC) Study: design and objectives. The ARIC investigators. *Am J Epidemiol* **129**, 687-702 (1989).
4. Fried, L.P. *et al.* The Cardiovascular Health Study: design and rationale. *Ann Epidemiol* **1**, 263-76 (1991).
5. Feinleib, M., Kannel, W.B., Garrison, R.J., McNamara, P.M. & Castelli, W.P. The Framingham Offspring Study. Design and preliminary data. *Prev Med* **4**, 518-25 (1975).
6. Splansky, G.L. *et al.* The Third Generation Cohort of the National Heart, Lung, and Blood Institute's Framingham Heart Study: design, recruitment, and initial examination. *Am J Epidemiol* **165**, 1328-35 (2007).
7. Williams, R.R. *et al.* NHLBI family blood pressure program: methodology and recruitment in the HyperGEN network. Hypertension genetic epidemiology network. *Ann Epidemiol* **10**, 389-400 (2000).
8. Arnett, D.K. *et al.* Genetic variation in NCAM1 contributes to left ventricular wall thickness in hypertensive families. *Circ Res* **108**, 279-83 (2011).
9. Coon, H. *et al.* Genome-wide linkage analysis of lipids in the Hypertension Genetic Epidemiology Network (HyperGEN) Blood Pressure Study. *Arterioscler Thromb Vasc Biol* **21**, 1969-76 (2001).
10. Bell, B., Rose, C.L. & Damon, A. The Veterans Administration longitudinal study of healthy aging. *Gerontologist* **6**, 179-84 (1966).
11. Lind, L., Fors, N., Hall, J., Marttala, K. & Stenborg, A. A comparison of three different methods to evaluate endothelium-dependent vasodilation in the elderly: the Prospective Investigation of the Vasculature in Uppsala Seniors (PIVUS) study. *Arterioscler Thromb Vasc Biol* **25**, 2368-75 (2005).
12. Friedewald, W.T., Levy, R.I. & Fredrickson, D.S. Estimation of the concentration of low-density lipoprotein cholesterol in plasma, without use of the preparative ultracentrifuge. *Clin Chem* **18**, 499-502 (1972).
13. Moayyeri, A., Hammond, C.J., Hart, D.J. & Spector, T.D. The UK Adult Twin Registry (TwinsUK Resource). *Twin Res Hum Genet* **16**, 144-9 (2013).
14. Design of the Women's Health Initiative clinical trial and observational study. The Women's Health Initiative Study Group. *Control Clin Trials* **19**, 61-109 (1998).
15. Anderson, G.L. *et al.* Implementation of the Women's Health Initiative study design. *Ann Epidemiol* **13**, S5-17 (2003).
