# Supplementary material for: A multi-ethnic epigenome-wide association study of leukocyte DNA methylation and blood lipids
Source: Nat Commun. 2021 Jun 28;12:3987. doi: 10.1038/s41467-021-23899-y (PMC8238961; doi:10.1038/s41467-021-23899-y)
Supplement: Supplementary file 3 — Description of Additional Supplementary Files [file 41467_2021_23899_MOESM3_ESM.pdf]

Description of Additional Supplementary Files

Supplementary Data 1:
DNA methylation and genotyping measurement details by cohort

Supplementary Data 2:
Significant CpG-lipid association meta-analyses and annotations for Models 3 and 4 stratified by racial/ethnic group and combined

Supplementary Data 3:
Significant methylation quantitative trait loci identified for the 30 CpGs found to be significantly associated with lipids in more than one racial/ethnic group

Supplementary Data 4:
Methylation quantitative trait loci (mQTL) entries in the methylation QTL database (http://www.mqtldb.org/) for seven CpGs found to have one or more mQTLs among Europeans in this study

Supplementary Data 5:
List of genome wide significant SNP-association for lipids identified through prior GWAS found to be within 10 megabases of significant CpG-lipid pairs identified in this epigenome wide association study without actually being located within the same gene.
