# Supplementary material for: A multi-ethnic epigenome-wide association study of leukocyte DNA methylation and blood lipids
Source: Nat Commun. 2021 Jun 28;12:3987. doi: 10.1038/s41467-021-23899-y (PMC8238961; doi:10.1038/s41467-021-23899-y)
Supplement: Supplementary file 9 — Reporting summary [file 41467_2021_23899_MOESM9_ESM.pdf]

nature research | reporting summary

nature research

Corresponding author(s): Themistocles L. Assimes

Last updated by author(s): Mar 28, 2021

Reporting Summary

Nature Research wishes to improve the reproducibility of the work that we publish. This form provides structure for consistency and transparency in reporting. For further information on Nature Research policies, see Authors & Referees and the Editorial Policy Checklist .

Statistics

For all statistical analyses, confirm that the following items are present in the figure legend, table legend, main text, or Methods section.

n/a	Confirmed	
☐	☒	The exact sample size (*n*) for each experimental group/condition, given as a discrete number and unit of measurement
☐	☒	A statement on whether measurements were taken from distinct samples or whether the same sample was measured repeatedly
☐	☒	The statistical test(s) used AND whether they are one- or two-sided<br>*Only common tests should be described solely by name; describe more complex techniques in the Methods section.*
☐	☒	A description of all covariates tested
☐	☒	A description of any assumptions or corrections, such as tests of normality and adjustment for multiple comparisons
☐	☒	A full description of the statistical parameters including central tendency (e.g. means) or other basic estimates (e.g. regression coefficient) AND variation (e.g. standard deviation) or associated estimates of uncertainty (e.g. confidence intervals)
☐	☒	For null hypothesis testing, the test statistic (e.g. *F*, *t*, *r*) with confidence intervals, effect sizes, degrees of freedom and *P* value noted<br>*Give P values as exact values whenever suitable.*
☒	☐	For Bayesian analysis, information on the choice of priors and Markov chain Monte Carlo settings
☒	☐	For hierarchical and complex designs, identification of the appropriate level for tests and full reporting of outcomes
☐	☒	Estimates of effect sizes (e.g. Cohen's *d*, Pearson's *r*), indicating how they were calculated

Our web collection on statistics for biologists contains articles on many of the points above.

Software and code

Policy information about availability of computer code

Data collection	The Illumina® Infinium HumanMethylation450 BeadChip and the Illumina BeadXpress reader with Illumina GenomeStudio® Methylation Module was used for DNA methylation data.
Data analysis	R (V.3.4.4 and later, https://www.r-project.org/) including minfi, wateRmelon, and qqman packages; Python 2.7.5 (https://www.python.org/downloads/); METASOFT (V.2.0.1, http://genetics.cs.ucla.edu/meta_jemdoc/). All the softwares are freely available through the links.

For manuscripts utilizing custom algorithms or software that are central to the research but not yet described in published literature, software must be made available to editors/reviewers. We strongly encourage code deposition in a community repository (e.g. GitHub). See the Nature Research guidelines for submitting code & software for further information.

Data

Policy information about availability of data

All manuscripts must include a data availability statement. This statement should provide the following information, where applicable:
- Accession codes, unique identifiers, or web links for publicly available datasets
- A list of figures that have associated raw data
- A description of any restrictions on data availability

The methylation QTL association results as well as the full summary statistics for the meta-analysis of the epigenome wide association study performed within each and across all racial/ethnic groups combined for all four models and all three lipid traits are available at :[https://doi.org/10.5061/dryad.qfttdz0d8].

Field-specific reporting

Please select the one below that is the best fit for your research. If you are not sure, read the appropriate sections before making your selection.

- [x] Life sciences
- [ ] Behavioural & social sciences
- [ ] Ecological, evolutionary & environmental sciences

For a reference copy of the document with all sections, see nature.com/documents/nr-reporting-summary-flat.pdf

Life sciences study design

All studies must disclose on these points even when the disclosure is negative.

Sample size	A total of 15 cohorts (N=16,265) from the epigenetics working group in the Cohorts for Heart and Aging Research in Genomic Epidemiology (CHARGE) consortium participated in this study. The total number of cohorts in the European, African, and Hispanic study populations is 12 (N=11,114), 9 (N=4,452), and 2 (N=699), respectively. We believe our study remains the single largest multi-ethnic epigenome-wide association study (EWAS) of blood lipids.
Data exclusions	We did not infer LDL in subjects with triglycerides > 400 mg/dL and we excluded lipid measures from subjects who did not fast for at least 8 hours as these circumstances are known to lead to unreliable estimates of LDL and triglycerides. Other pre specified exclusion criteria included outliers as defined by >5 standard deviations from the mean of a blood lipid in each cohort as these may be technical artifacts. To maximize the reliability of the CpG methylation measures, any single value with a detection p-value > 0.01 was set to missing. In each cohort, we excluded probes with a detection p-value > 0.01 in greater than 5% of samples. In addition, we excluded samples with a detection p-value > 0.01 in greater than 5% of probes. To avoid spurious signals in DNA methylation data, we excluded 29,233 CpGs that co-hybridize to alternate genomic sequences (highly homologous to the intended targets).
Replication	A total of 30 CpG-lipid trait associations were found to be significant in more than one racial/ethnic group European population (N=11,114) were replicated in African (N=4,452) and Hispanic (N=699) populations. The remaining association were not found to be significant in more than one racial/ethnic group. This could be due to lower statistical power from smaller sample sizes and race/ethnic specific differences.
Randomization	Our study is observational study with both blood lipid and DNA methylation levels measured at the same time point (cross-sectional). So we did not apply any randomization.
Blinding	Our study is observational study and there was no blinding.

Reporting for specific materials, systems and methods

We require information from authors about some types of materials, experimental systems and methods used in many studies. Here, indicate whether each material, system or method listed is relevant to your study. If you are not sure if a list item applies to your research, read the appropriate section before selecting a response.

Materials & experimental systems

n/a	Involved in the study	
[x]	[ ]	Antibodies
[x]	[ ]	Eukaryotic cell lines
[x]	[ ]	Palaeontology
[x]	[ ]	Animals and other organisms
[ ]	[x]	Human research participants
[x]	[ ]	Clinical data

Methods

n/a	Involved in the study	
[x]	[ ]	ChIP-seq
[x]	[ ]	Flow cytometry
[x]	[ ]	MRI-based neuroimaging

Human research participants

Policy information about studies involving human research participants

Population characteristics	We analyzed 12 cohorts of Europeans (EA) involving 11,114 participants, 7 cohorts of African Americans (AA) involving 4,425, and 2 cohorts of Hispanics (HISP) involving 699 participants (Table 1, Supplementary methods). The TwinsUK, WHI-BA23, and WHI-EMPC cohorts were composed of female participants only while NAS was composed of male participants only. The range of mean age, body mass index (BMI), high-density lipoprotein (HDL) levels, low-density lipoprotein (LDL) levels, and triglyceride (TG) levels was 42.7 to 76.0 year, 26.6 to 32.6 kg/m2, 45.5 to 59.3 mg/dl, 104.9 to 152.6 mg/dl, 74.1 to 168.5 mg/dl, respectively (Table 1). The percentage of study participants taking any lipid control medication at time of blood lipid measurement ranged between 0% in the Amish to 44% in FHS.
Recruitment	A total of 15 cohorts (N=16,265) from the epigenetics working group in the Cohorts for Heart and Aging Research in Genomic Epidemiology (CHARGE) consortium participated in this study. These included the Old Order Amish (OOA), Atherosclerosis Risk in Communities (ARIC), Bogalusa Heart Study (BHS), Cardiovascular Health Study (CHS), Framingham Heart Study (FHS), Genetic Epidemiology Network of Arteriopathy (GENOA), Genetics of Lipid Lowering Drugs and Diet Network (GOLDN), Hypertension Genetic Epidemiology Network (HyperGEN), Cooperative health research in the Region of Augsburg (KORA), Normative Aging Study (NAS), Prospective Investigation of Vascularity of Uppsala Elders Study (PIVUS), Rotterdam Study (RS), UK Adult Twin Registry (TwinsUK), Women's Health Initiative Broad Agency Announcement 23 (WHI-BA23), and the Women's Health Initiative Epigenetic Mechanisms of PM-Mediated CVD (WHI-EMPC) cohorts. Four cohorts, BHS, CHS, WHI-BA23, and WHI-EMPC, examined more than one race/ethnic group. The total number of cohorts in the European, African, and Hispanic study populations is 12 (N=11,114), 9 (N=4,452), and 2 (N=699), respectively. The participating cohorts are described in the Supplementary materials.
Ethics oversight	Each pf the 15 cohorts included secured local IRB approval for this study. Statement is included in main text and please see supplementary methods for more details by cohort.

Note that full information on the approval of the study protocol must also be provided in the manuscript.
